# Innovative Implementation Strategies for Familial Hypercholesterolemia Cascade Testing: The Impact of Genetic Counseling

**DOI:** 10.3390/jpm14080841

**Published:** 2024-08-09

**Authors:** Kelly M. Morgan, Gemme Campbell-Salome, Nicole L. Walters, Megan N. Betts, Andrew Brangan, Alicia Johns, H. Lester Kirchner, Zoe Lindsey-Mills, Mary P. McGowan, Eric P. Tricou, Alanna Kulchak Rahm, Amy C. Sturm, Laney K. Jones

**Affiliations:** 1 Department of Genomic Health, Research Institute, Geisinger, 100 N. Academy Avenue, Danville, PA 17922, USA; gcampbell3@geisinger.edu (G.C.-S.); nlwalters1@geisinger.edu (N.L.W.); ambrangan@geisinger.edu (A.B.); ztlindseymills@geisinger.edu (Z.L.-M.); amycs@23andme.com (A.C.S.);; 2 Department of Population Health Sciences, Research Institute, Geisinger, 100 N. Academy Avenue, Danville, PA 17922, USA; hlkirchner@geisinger.edu; 3 WellSpan Health, 605 S. George Street, York, PA 17401, USA; mbetts2@wellspan.org; 4 Biostatistics Core, Research Institute, Geisinger, 100 N. Academy Avenue, Danville, PA 17922, USA; amjohns1@geisinger.edu; 5 Family Heart Foundation, 605 E. Colorado Blvd Ste 180, Pasadena, CA 91101, USAet@familyheart.org (E.P.T.); 6 Heart and Vascular Institute, Geisinger, 100 N. Academy Avenue, Danville, PA 17922, USA; 7 23andMe, 223 N. Mathilda Avenue, Sunnyvale, CA 94086, USA

**Keywords:** genetic counseling, cascade testing, familial hypercholesterolemia, population genomic screening

## Abstract

The IMPACT-FH study implemented strategies (packet, chatbot, direct contact) to promote family member cascade testing for familial hypercholesterolemia (FH). We evaluated the impact of genetic counseling (GC) on medical outcomes, strategy selection, and cascade testing. Probands (i.e., patients with FH) were recommended to complete GC and select sharing strategies. Comparisons were performed for both medical outcomes and strategy selection between probands with or without GC. GEE models for Poisson regression were used to examine the relationship between proband GC completion and first-degree relative (FDR) cascade testing. Overall, 46.3% (81/175) of probands completed GC. Probands with GC had a median LDL-C reduction of −13.0 mg/dL (−61.0, 4.0) versus −1.0 mg/dL (−16.0, 17.0) in probands without GC (*p* = 0.0054). Probands with and without GC selected sharing strategies for 65.3% and 40.3% of FDRs, respectively (*p* < 0.0001). Similarly, 27.1% of FDRs of probands with GC completed cascade testing, while 12.0% of FDRs of probands without GC completed testing (*p* = 0.0043). Direct contact was selected for 47 relatives in total and completed for 39, leading to the detection of 18 relatives with FH. Proband GC was associated with improved medical outcomes and increased FDR cascade testing. Direct contact effectively identified FH cases for the subset who participated.

## 1. Introduction

Familial hypercholesterolemia (FH) is one of the most common inherited cardiovascular conditions [1]. Recognizing and treating FH early can prevent severe outcomes such as atherosclerotic cardiovascular disease (ASCVD) [2,3]. It is estimated that only approximately 10% of individuals with FH have been diagnosed [4]. Genetic counselors, with specialized knowledge in the medical and psychosocial aspects of genetic disease, have an important role in the identification and management of FH. Through risk assessment, coordination of genetic testing services, and education on the clinical implications of FH, genetic counselors prompt action for probands (the first family member diagnosed) and their at-risk relatives to address FH [5,6].

Genetic counseling (GC) can improve adherence to medical management recommendations and medical outcomes [7]. Traditionally, GC is provided before and after genetic testing (i.e., pre- and post-test GC). Population genomic screening followed by GC (post-result GC) is an emerging model that promotes increased access to genetic testing [8]. Consistent with traditional GC, population genomic screening post-result GC is associated with the completion of recommended risk management for conditions including FH [9]. However, overall engagement with GC following the receipt of population genomic screening results remains variable, with roughly half of participants completing GC in one such model [10].

Cascade testing, or targeted family member testing, can substantially increase the detection of FH [11,12]. The current standard of care in the United States (U.S.) places the responsibility of family notification on the proband, which contributes to cascade testing barriers [13,14]. To address this, novel approaches to support family communication, including more active involvement of genetic counselors in the care and testing of at-risk relatives, are being explored [15,16,17]. Cascade testing programs for FH that facilitate direct contact of at-risk relatives by genetic counselors or other genetics specialists are established outside of the U.S. and are beginning to emerge within the U.S. healthcare system [11,18]. These programs have been successful in identifying new cases of FH, particularly for families with a genetic diagnosis [19].

As novel models to improve the identification of individuals with genomic risk emerge, it is important to understand the role of genetic counselors. The implementation of a cascade testing intervention for FH within a population genomic screening program with recommended post-result GC provides the opportunity to examine genetic counselor’s impact on the outcomes of probands and at-risk relatives [20]. We explored factors associated with proband completion of GC appointments and compared probands who did or did not complete a GC appointment for proband medical behaviors and outcomes, family communication decisions, and cascade testing completion. Furthermore, we determined the uptake and outcomes for relatives following their participation in a direct contact program facilitated by genetic counselors, regardless of whether the proband selecting this sharing strategy had GC or not.

## 2. Materials and Methods

The Identification Methods, Patient Activation, and Cascade Testing for FH (IMPACT-FH) study designed and implemented a prospective pragmatic study of innovative strategies to support family communication and cascade testing, specifically a Family and Healthcare Professional Packet (packet), Family Sharing and Cascade Chatbots (chatbots), and the FH Outreach and Support Program (direct contact) (Geisinger IRB 2020-0579) [20]. Complete study methods are described elsewhere, and information relevant to these analyses is included below [21].

### 2.1. Study Setting

The IMPACT-FH study was conducted within a population genomic screening context. The MyCode^®^ Genomic Screening and Counseling (GSC) program returns clinically actionable genetic results, including FH, to individuals enrolled in Geisinger’s MyCode Community Health Initiative, a health-system-wide biobank [8,22]. Probands with an FH result (i.e., likely pathogenic or pathogenic variant in *LDLR*, *APOB*, or *PCSK9*) disclosed by a genetic counselor through the GSC program between July 2021 and April 2022 who met IMPACT-FH eligibility criteria were enrolled in the study [21]. IMPACT-FH probands were offered the IMPACT-FH strategies to communicate their FH results with their relatives [20]. These implementation strategies were introduced to probands at the time of the initial MyCode results disclosure and revisited at study follow-up timepoints and GC.

### 2.2. Genetic Counseling Procedures

**Figure 1:**
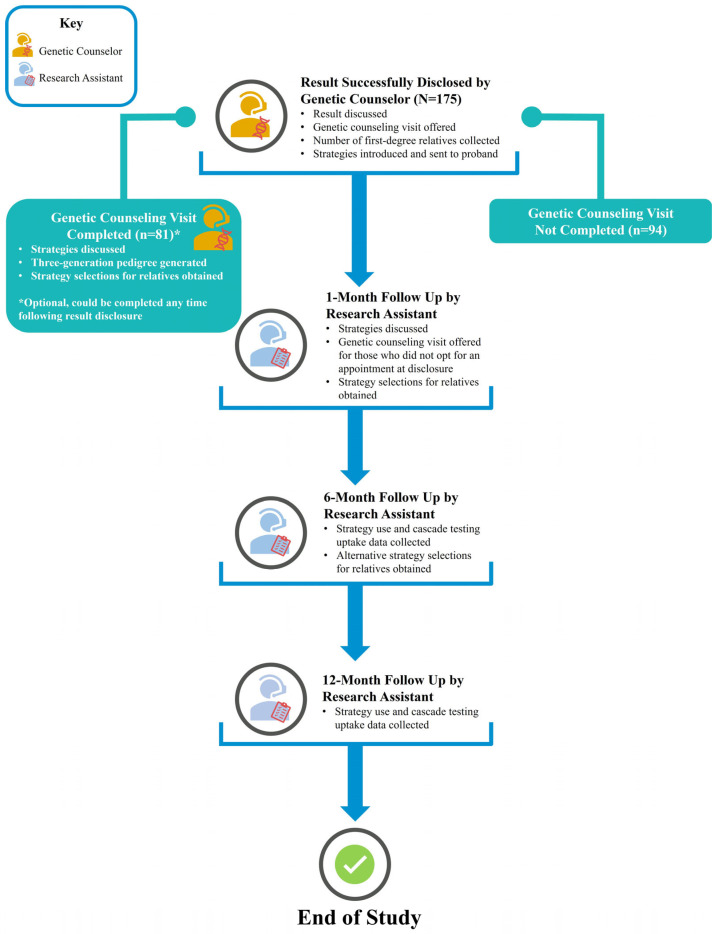
Overview of Results Disclosure, Genetic Counseling, and Study Follow-up.

IMPACT-FH probands were offered a GC appointment following the same clinical processes as the MyCode GSC. Probands who receive a MyCode result are recommended to complete, but do not always opt for, a GC appointment. These post-result GC appointments are available virtually or in-person at no cost through the MyCode GSC program [8]. These appointments are tailored to this genotype-first approach, incorporating all relevant aspects from both pre- and post-test GC, including discussion of cascade testing, into a one-visit model [8]. A GC appointment is offered at the time of results disclosure, and for those who do not opt for an appointment, at a 1-month follow-up timepoint (Figure 1).

### 2.3. Implementation Strategies

The IMPACT-FH implementation strategies are described in detail elsewhere [20]. Briefly, the packet includes detailed written information about the proband’s test result and genetic testing options for relatives and relative’s healthcare providers [20]. The Family Sharing Chatbot (FSC) can be utilized by probands to send the Cascade Chatbot (CC) to their relatives. Relatives can use the CC to learn about FH and consent to cascade testing. Through direct contact, a genetic counselor contacts an at-risk relative and/or an at-risk relative’s clinician directly, at the request of and with verbal HIPAA authorization from the proband. Following a “primer” letter sent to relatives with the option to opt out, a genetic counselor makes up to three outreach attempts to relatives and/or relatives’ health care providers. For relatives who were in-state (PA), genetic testing could be ordered during direct contact. For out-of-state relatives, options to complete testing locally were provided, including the option to order a mail-in genetic testing kit through the CC. Probands, or their designee, could select one or more sharing strategies for each of their adult (age ≥ 18 years old) relatives at multiple timepoints: results disclosure, GC appointment, 1-month follow-up, and 6-month follow-up. Strategies were not selected for minors (<18); however, these individuals were eligible for cascade testing.

### 2.4. Identification of At-Risk Relatives

The number of first-degree relatives (FDRs) was collected for all probands at disclosure. Additionally, the study collected names, date of birth/age, and degree of relationship for any of the proband’s living blood relatives that probands selected a strategy for and/or reported completed cascade testing at any study timepoint. For probands who opted to complete a GC appointment, a family tree (pedigree) documenting relatives through the third-degree of the proband was generated (Figure 1). FDRs were defined as relatives who were parents, full siblings, and adult children (age ≥ 18). All relatives were defined as all living adult blood relatives (including FDRs) (age ≥ 18). Minors were any blood relative less than the age of 18 years old.

### 2.5. Study Follow-Up Timepoints

As described in detail elsewhere, all probands were contacted by the study team at 1-month, 6-month, and 12-month post-results return [21]. The 1-month follow-up timepoint included strategy selection and the opportunity to re-send any implementation strategies upon request. At the 6-month follow-up timepoint, outcomes of family communication choices (i.e., was the strategy sent) and cascade testing uptake were collected, and additional strategy selection for untested relatives was encouraged. The final 12-month follow-up timepoint included proband-reported outcomes on family communication, strategy use, and cascade testing uptake. For probands who opted to complete a GC appointment, this offered an additional opportunity to discuss and collect the proband’s implementation strategy selections (Figure 1).

### 2.6. Outcomes

#### 2.6.1. Proband Demographics and GC Completion

Proband demographic information and the completion of the GC were collected by the study team. In families where multiple individuals received an FH result from the MyCode GSC (9 families total), each of these individuals was counted as a proband.

#### 2.6.2. Proband Medical Behaviors and Outcomes

Proband pre- and post-disclosure medical behaviors and outcomes were obtained via data extraction from the electronic health records by a data analyst. Data extracted included: new and follow-up cardiology appointment completion, lipid lowering therapies (LLTs) prescribed, lipid panel completion, average low-density lipoprotein cholesterol (LDL-C) levels, and cardiovascular event occurrence (stroke, myocardial infarction, and/or coronary artery disease). Probands who withdrew from the MyCode study following results disclosure (*n* = 2) or were deceased within 1 year of results return (*n* = 3) were not included in this prospective data pull. All probands had at least 12 months of follow-up from results receipt to the time of analysis. The medical behaviors and outcomes of probands who did or did not complete a GC appointment were compared.

#### 2.6.3. Family Communication Choice

Family communication choice was defined as the selection of implementation strategies (packet, chatbot, or direct contact) by the proband to share their results with their relatives. Family communication choices for FDRs of probands who did or did not complete a GC appointment were compared. Family communication choices for all relatives were also reported.

#### 2.6.4. Cascade Testing Uptake

Cascade testing was defined as relatives completing cholesterol and/or genetic testing. Data on cascade testing was collected through proband reports at study follow-up timepoints or optional study interviews, relative self-reports during optional study interviews, from Geisinger directly, and from the genetic testing laboratory for relatives tested outside of Geisinger who provided consent/authorization. Cascade testing uptake at 12-months by FDRs of probands with or without GC was compared. Cascade testing uptake for all relatives and minors was also reported.

#### 2.6.5. Direct Contact

Uptake and outcomes of direct contact (completion of direct contact, completion of genetic cascade testing, and genetic test result) were collected by the study.

### 2.7. Statistical Analysis

All data for probands were fully described using medians and inter-quartile ranges (IQRs) for continuous variables and frequency and percentage for categorical variables. Demographic and pre- and post-disclosure proband medical behavior was compared between those probands who completed and did not complete GC using Pearson’s Chi-Square and the Wilcoxon rank sum non-parametric tests, as appropriate. Descriptive statistics were provided for family communication choice for all relatives broken down by proband GC. For FDRs, family communication choice variables were compared between proband GC status using Pearson’s Chi-Square, Fisher’s Exact, and Wilcoxon rank sum tests. Analyses at the FDR level were accomplished using Generalized Estimating Equations (GEEs) models to account for the correlation due to clustering per proband, and an independent correlation structure was used. A GEE model for Poisson regression was utilized to examine cascade testing in FDR by proband GC, and incident rate ratios were reported. Additionally, descriptive statistics were reported for cascade testing in all relatives by proband GC. Relatives under the age of 18 (minors) and non-blood relatives were excluded from the primary analyses.

## 3. Results

### 3.1. Proband Demographics and GC Completion

The IMPACT-FH study included 175 probands who received a likely pathogenic or pathogenic variant in the *APOB* (85/175, 48.6%) or *LDLR* (90/175, 51.4%) gene. Overall, 46.3% (81/175) of probands completed a GC appointment, and 53.7% (94/175) of probands did not complete a GC appointment following results disclosure. Probands who minimized contact with the study were less likely to complete a GC appointment (*p* = 0.0205). Probands in both groups reported a median of 4 FDRs to the IMPACT-FH team. Probands with GC reported a median of 16 total relatives to the IMPACT-FH team, while probands without GC reported a median of 5 total relatives to the IMPACT-FH team (*p* < 0.0001) (Table 1).

### 3.2. Proband Medical Behaviors and Outcomes

Before results disclosure, there were no significant differences in completion of cardiology appointment(s), LLT prescriptions, completion of lipid panels, average LDL-C levels, or cardiovascular events (stroke, myocardial infarction, and/or coronary artery disease) between probands who would go on to complete or not complete a GC appointment. Following disclosure, probands who completed a GC appointment were more likely to complete a new or return cardiology appointment (*p* < 0.0001), have a lipid panel (*p* = 0.0008), and have an active LLT prescription (*p* = 0.0063). As a result, probands with GC had a median LDL-C reduction of −13.0 mg/dL (IQR: −61.0, 4.0), while probands without GC had a median LDL-C reduction of −1.0 mg/dL (IQR: −16.0, 17.0) (*p* = 0.0054). Probands with GC had a median post-disclosure LDL-C of 100.0 mg/dL (IQR: 64.0, 128.0), while probands without GC had a median LDL-C of 118.0 mg/dL (IQR: 81.0, 150.0) after disclosure (*p* = 0.0473). There were no significant differences in post-disclosure cardiovascular events between probands who did and did not complete GC (Table 2).

### 3.3. Family Communication Choice

Out of 81 probands who completed GC, they collectively selected 453 strategies. In this group, the packet, chatbot, and direct contact were selected 270, 143, and 40 times, respectively. Out of 94 probands who did not complete GC, they collectively selected 210 strategies. In this group, the packet, chatbot, and direct contact were selected 158, 45, and 7 times, respectively.

#### 3.3.1. First-Degree Relatives

At least one strategy was selected for 65.3% (224/343) of FDRs of probands with GC, while at least one strategy was selected for 40.3% (154/382) of FDRs of probands without GC (*p* < 0.001). An average of 3.6 (SD: 1.97) FDRs per proband with GC had a strategy selected, while an average of 2.9 (SD: 1.44) FDRs per proband without GC had a strategy selected (*p* = 0.0005). There was no significant difference in the number of strategies selected per relative between relatives of probands with or without GC, with a median of 1 strategy selected per relative in both groups. Certain strategies were more likely to be selected by probands with GC: chatbot only (*p* < 0.0001), direct contact only (*p* = 0.0209), packet and chatbot (*p* = 0.0053), and packet and direct contact (*p* = 0.0209) (Table 3).

#### 3.3.2. All Relatives

At least one strategy was selected for 25.5% (367/1440) of all relatives of probands with GC, while at least one strategy was selected for 36.6% (174/475) of all relatives of probands without GC. An average of 5.9 (SD: 5.37) relatives per proband with GC had a strategy selected, while an average of 3.1 (SD: 1.58) relatives per proband without GC had a strategy selected. A median of 1 strategy was selected per relative in both groups (Table 4).

### 3.4. Cascade Testing Uptake

#### 3.4.1. First-Degree Relatives

Cascade testing (lipid and/or genetic testing) was completed in 27.1% (93/343) of FDRs of probands with GC, compared to 12.0% (46/382) of FDRs of probands without GC (*p* = 0.0043). Genetic testing only was completed in 7.0% (24/343) of FDRs of probands with GC, compared to 1.6% (6/382) of FDRs of probands without GC (*p* = 0.0066). Lipid testing only was completed by 15.2% (52/343) of FDRs of probands with GC, compared to 9.4% (36/382) of FDRs of probands without GC, which was not significantly different between groups (*p* = 0.1634). Similarly, both genetic and lipid testing was completed by 5.0% (17/343) of FDRs of probands with GC, compared to 1.0% (4/382) of FDRs of probands without GC (*p* = 0.0695) (Table 5). 

#### 3.4.2. All Relatives

Cascade testing (lipid and/or genetic testing) was completed in 8.5% (123/1440) of all relatives of probands with GC and 11.4% (54/475) of all relatives of probands without GC. Genetic testing only was completed in 2.7% (39/1440) of all relatives of probands with GC and 1.9% (9/475) of all relatives of probands without GC. Lipid testing only was completed by 4.5% (65/1440) of all relatives of probands with GC and 8.4% (40/475) of all relatives of probands without GC. Both genetic and lipid testing was completed by 1.3% (19/1440) of all relatives of probands with GC and 1.1% (5/475) of all relatives of probands without GC (Table 6).

#### 3.4.3. Minors

Of the 163 minor relatives of probands, 27.5% (36/131) of those related to probands with GC completed cascade testing (8 both, 3 lipid testing only, 25 genetic testing only), while 15.6% (5/32) of those related to probands without GC completed cascade testing (0 both, 3 cholesterol testing only, 2 genetic testing only). For minor relatives with genetic testing completed, the most common ways to access testing were: a pediatric cascade GC appointment at Geisinger or in partnership with a local community center (51.4%, 18/35) or during a parent’s GC appointment or direct contact (34.3%, 12/35). Other ways that genetic testing was completed were through secondary finding/reanalysis of whole exome testing for other indications (8.6%, 3/35) or with an external primary care provider (5.7%, 2/35).

### 3.5. Direct Contact

**Figure 2:**
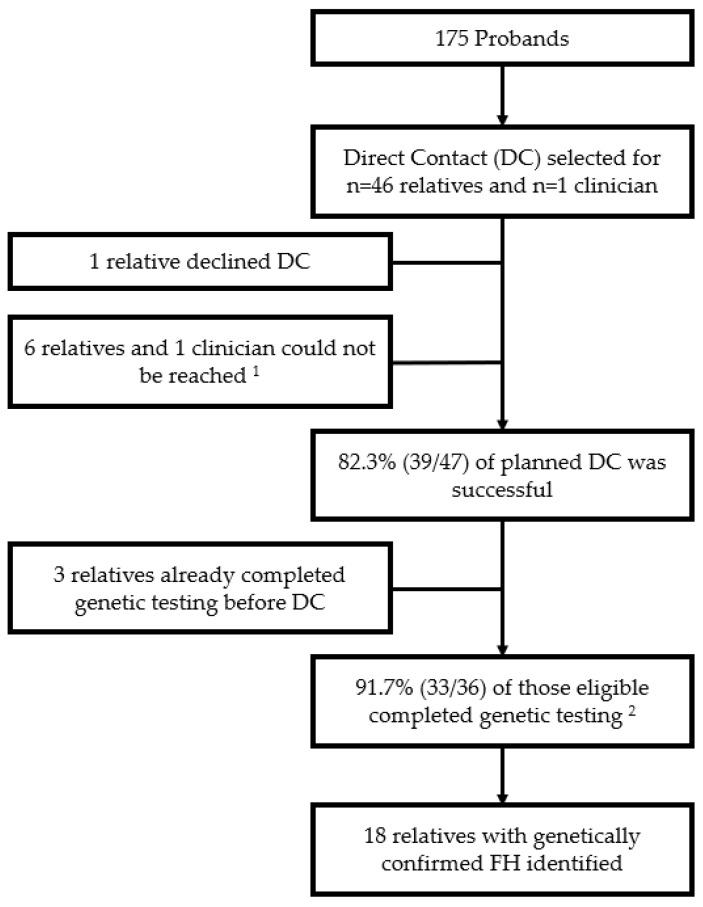
Direct Contact Uptake and Outcomes. ^1^ Three outreach attempts; ^2^ Two relatives declined genetic testing during DC, and one relative did not complete the genetic testing that was ordered during DC.

Direct contact was selected for 46 relatives and one relative’s clinician, resulting in 14 participating families. Within each family, direct contact was selected for a range of 1–9 relatives, with a median of 2.5 relatives. While the majority (33/47, 70.2%) of relatives were FDRs of the proband, there were seven second-degree (14.9%) and seven third-degree (14.9%) relatives. Most relatives (41/47, 87.2%) resided in-state (PA); however, six relatives (12.8%) were out-of-state (VA, NJ, NC, FL, NY, and CA). One relative declined direct contact, and seven relatives/their clinician were *unable to be* reached. Overall, 39 direct contacts were successfully completed. These calls had a mean duration of 16 min. A total of three relatives had genetic testing before direct contact, resulting in 36 relatives eligible for genetic testing during direct contact. Among these 36 relatives, genetic testing was ordered by the *IMPACT-FH* team for the majority (30/36, 83.3%), with only two relatives declining genetic testing during direct contact. Alternate ways to complete genetic testing—referral to a local genetic counselor (*n* = 1) or study chatbot (*n* = 3)—were chosen by out-of-state relatives. Overall, 91.7% (33/36) of relatives eligible to complete genetic testing following direct contact did so, and 18 relatives with FH were identified (Figure 2).

## 4. Discussion

The IMPACT-FH study evaluated a novel and multifaceted approach that improved cascade testing uptake in families with FH detected through population genomic screening [21]. The current study demonstrates the importance of GC within IMPACT-FH across probands’ medical behaviors and outcomes, family communication, cascade testing uptake, and facilitation of direct contact. These findings can inform future implementation strategies to improve cascade testing for FH and highlight important considerations regarding the role of GC in population genomic screening. 

For probands, we found that completion of GC was significantly associated with medical management adherence (completion of a cardiology appointment, having a lipid panel, and taking LLTs), which resulted in significant differences in LDL-C levels compared to individuals who did not complete GC post-result. This expands on previous work demonstrating that GC was associated with the completion of one or more recommended medical management procedures for probands with an FH result identified through MyCode [9]. The median follow-up LDL-C of 100.0 mg/dL in probands with GC compared to 118.0 mg/dL in probands without GC in IMPACT-FH has clinical implications. Patients with FH are recommended to have an LDL-C of at least < 100 mg/dL, if no coronary artery disease or other risk factors are present, to reduce the risk of cardiovascular events [23]. While there was no significant difference in cardiovascular outcomes at the 1-year follow-up timepoint, we hypothesize that a divergence in clinical outcomes could emerge over a longer follow-up period as a result of the difference in LDL-C levels between groups. These findings highlight the importance of GC for probands receiving genomic screening findings. 

The benefits of completing GC extend beyond probands to improve FH identification for at-risk relatives. For FDRs, we found that probands with GC selected at least one strategy for 65.3% of FDRs, compared to 40.3% of FDRs of probands without GC. GC provided an additional timepoint for strategy selection. Beyond this, genetic counselors aim to facilitate family communication through thorough family history collection, education about the implications of test results for family members, and the provision of psychosocial and logistical support for family sharing [5,24]. Decisions regarding the type of strategy selected were also significant. Results from the overall study demonstrate that among the IMPACT-FH strategies, the chatbot and direct contact were more effective than the packet at facilitating cascade testing, and direct contact specifically resulted in 16.78 higher odds of cascade testing completion compared to no strategy [21]. We found that the chatbot and direct contact, on their own or in addition to the packet, were more likely to be selected by probands with GC. With respect to direct contact selection, we hypothesize that the trust established between the GC and proband during GC may increase the proband’s comfort with the GC reaching out to their relatives through direct contact; however, overall uptake of direct contact was low (*n* = 47). Qualitative analysis of the study reveals that direct contact was rated as the most acceptable and appropriate strategy and suggests that reliance on the proband to select strategies for their relatives, including direct contact, may be a barrier to uptake [25]. 

The finding that FDRs of probands with GC were more likely to complete cascade testing, with 27.1% of FDRs of probands with GC tested compared to 12.0% of relatives of probands without GC tested, demonstrates the impact of GC on cascade testing uptake in this program. The IMPACT-FH study enhanced the clinical tools available to genetic counselors. Through genetic counselor facilitation of strategy selection, more relatives of probands with GC learned about the familial result and completed cascade testing. Among cascade testing options, the finding that genetic testing but not lipid testing was significantly more likely to be completed in FDRs of probands with GC is likely due to the comprehensive education provided in GC. Specifically, genetic counselors explain the benefits and limitations of different testing options, including the benefits of a definitive genetic diagnosis, such as improved insurance coverage for certain LLTs [5]. 

Comparing outcomes between relatives beyond FDRs is limited by differences in extended family history data collection between groups. The finding that probands with GC reported a median of 16 total relatives compared to 5 relatives reported by probands without GC can largely be explained by the clinical collection of a 3-generation pedigree during GC. While we observed numerically more strategy selection and cascade testing in all relatives of probands with GC, we observed proportionally lower uptake of both of these outcomes (e.g., 123/1440 (8.5%) of all relatives of probands with GC completed cascade testing, while 54/475 (11.4%) of all relatives of probands without GC completed cascade testing). GC includes the identification of more relatives who are ultimately not at risk for FH (e.g., on the other side of the family, more extended relatives) and includes counseling about a step-wise approach to cascade testing, which may result in more targeted outreach to at-risk relatives. Further study with equivalent family history collection between groups is needed to understand the impact of GC on family communication and cascade testing uptake for more extended relatives participating in this model. 

Direct contact has been shown to be effective outside of the U.S. in the setting of centralized health systems and registries, which facilitate connections between providers and relatives [26,27,28,29,30,31]. IMPACT-FH implemented and evaluated one of the first direct contact programs for FH in the U.S. and deployed phone call outreach from a genetic counselor. Another study in the U.S. by Miller and colleagues utilized written direct contact outreach and found that this model successfully reached 51% (129/253) of relatives of probands with genetically identified FH, and 86% (111/129) of these relatives completed genetic testing [19,32]. Compared to this written direct contact strategy, we found that a written primer followed by a genetic counselor phone call led to higher rates of participation with 82.9% (39/47) of relatives reached by the genetic counselor and similar rates of genetic testing completion (91.7%) (33/36). Factors that may have contributed to a higher response rate in IMPACT-FH direct contact that should be considered in future direct contact program design include: offering direct contact amongst other options to facilitate choices about which scenarios and for which relatives/probands direct contact is best suited for; coaching probands to provide a “heads up” to their relatives about direct contact; phone call outreach; and completion of this phone call outreach by a genetic counselor specifically [20,33,34]. Importantly, IMPACT-FH direct contact overcame common barriers to familial communication and cascade testing by reaching distantly related (e.g., third-degree relatives) and geographically distant relatives. Nearly one-third of relatives for whom direct contact was selected were 2nd (e.g., aunts, uncles, grandparents) or 3rd degree (e.g., cousins) relatives of the proband. GC licensure laws limit the provision of GC and testing services for relatives who reside out of state from the proband. Specifically, the genetic counselor providing care must be licensed in the relative’s location [35]. Offering direct contact as part of a multi-component intervention expanded options for out-of-state relatives to complete testing with the opportunity to complete genetic testing through the chatbot following direct contact in addition to traditional referral to a local genetic counselor. As a result, 66.7% (4/6) of out-of-state relatives successfully completed genetic testing through follow-up initiated by direct contact.

The IMPACT-FH study uniquely delivered a multi-component intervention that facilitated choice among strategies [21]. These implementation strategies have each been integrated into GC practice to varying extents. Family letters, the most selected strategy across IMPACT-FH probands, are routinely used for patients undergoing GC for a range of indications [36,37]. Chatbots are increasingly being incorporated to support GC services [38]. However, the application of chatbots to facilitate cascade testing is novel to the IMPACT-FH study and MyCode GSC to our knowledge [21,39]. Direct contact has been explored for other genetic conditions [33,40,41] including a pilot study by Frey et al. where 70% of relatives reached via direct contact went on to complete genetic testing for the hereditary cancer result in their family [42]. Given the relevance of these strategies throughout GC practice, future work should consider how to adapt the IMPACT-FH approach to address the low rates of cascade testing reported across hereditary conditions [14,43].

Suboptimal uptake of GC remains a persistent barrier to effective genetic test result follow-up within population genomic screening. The uptake of GC for IMPACT-FH probands (46.3%) is consistent with the overall GC uptake of 47.5% across genetic test results in the MyCode GSC program [10]. In the IMPACT-FH cohort, unsurprisingly, probands who minimized contact (i.e., asked the study not to follow-up with them) were less likely to complete GC. In the broader MyCode cohort, probands who had more direct engagement with the program (i.e., were reached by a genetic counselor or genetic counseling assistant for phone disclosure of their result) were more likely to complete GC [10]. Schwartz et al. also identified other clinically important factors associated with increased uptake of GC not evaluated in this study, including having fewer comorbidities and a shorter travel distance to the clinic [10]. Given the importance of GC in guiding next steps for probands and their relatives in our work and others, interventions to improve uptake of GC are needed to fully realize the benefits of population genomic screening.

A limitation to this analysis, as described above, is the difference in family history data collection for extended relatives between groups, which limits the comparisons that can be made beyond FDRs. Furthermore, it is challenging to fully attribute the outcomes following GC to GC itself, despite similar medical behaviors between groups before GC, due to the possibility that probands who chose to complete GC were more motivated at baseline to follow-up on their result compared to probands who opted out of GC. Understanding and addressing these upstream attitudes is an important component of interventions to improve the uptake of GC. Additionally, there are established disparities in access to and completion of GC in the literature related to race and ethnicity that would not have been detected in our study population [44,45,46]. Cascade testing interventions that include a GC component must address these barriers to ensure equitable access to care. Lastly, this study did not investigate the sustainability of the cascade testing intervention. Future work should evaluate the resourcing and cost of this type of program, including the cost of each implementation strategy, genetic counselor, and other staff time required to deliver the program.

## 5. Conclusions

GC was a key component of a multi-level intervention to improve cascade testing uptake for FH. For probands, these results further confirm the clinical benefit of GC in the population genomic screening context, including meaningful differences in the clinical outcome of LDL-C levels. FDRs of probands who completed GC benefited from more communication about the FH result in the family and were more likely to complete cascade testing as a result. Genetic counselor direct contact was highly effective in facilitating cascade testing when selected. Taken together, these results indicate that future cascade testing programs should continue to incorporate GC services. Interventions to improve GC uptake are needed and expected to lead to clinical benefit for probands and their relatives. 

## Figures and Tables

**Table 1:** Proband Demographics and Completion of Genetic Counseling.

	IMPACT-FH Probands with GC (*n* = 81)	IMPACT-FH Probands without GC (*n* = 94)	*p*-Value
Vital Status, *n* (%)			0.6499 ^1^
Alive	80 (98.8%)	92 (97.9%)	
Deceased	1 (1.2%)	2 (2.1%)	
Proband Minimized Contact, *n*, (%)			0.0205 ^1^
No	78 (96.3%)	81 (86.2%)	
Yes	3 (3.7%)	13 (13.8%)	
Age at FH Result Disclosure			0.0935 ^2^
Median (IQR)	55.1 (43.0, 68.4)	61.8 (45.5, 70.7)	
Range	21.9, 86.4	21.1, 89.0	
Sex, *n* (%)			0.4737 ^1^
Male	31 (38.3%)	41 (43.6%)	
Female	50 (61.7%)	53 (56.4%)	
Number of FDRs			0.4870 ^2^
Median (IQR)	4.0 (3.0, 5.0)	4.0 (3.0, 5.0)	
Range	0.0, 13.0	0.0, 13.0	
Total Number of Relatives			<0.0001 ^2^
Median (IQR)	16.0 (12.0, 24.0)	5.0 (3.0, 5.0)	
Range	2.0, 43.0	1.0, 17.0	
Race, *n* (%)			0.2317 ^1^
White	80 (98.8%)	90 (95.7%)	
Other ^3^	1 (1.2%)	4 (4.3%)	
Ethnicity, *n* (%)			0.1048 ^1^
Non-Hispanic or Latino	81 (100.0%)	91 (96.8%)	
Other ^4^	0 (0.0%)	3 (3.2%)	
State, *n* (%)			0.2317 ^1^
Not PA	1 (1.2%)	4 (4.3%)	
PA	80 (98.8%)	90 (95.7%)	
Insurance Type, *n* (%)			0.6954 ^1^
No insurance	2 (2.5%)	3 (3.2%)	
Private insurance only	40 (49.4%)	37 (39.4%)	
Medicaid only	7 (8.6%)	11 (11.7%)	
Medicare only	4 (4.9%)	4 (4.3%)	
Private insurance and Medicaid	3 (3.7%)	1 (1.1%)	
Private insurance and Medicare	22 (27.2%)	35 (37.2%)	
Medicaid and Medicare	1 (1.2%)	2 (2.1%)	
Medicare and Tricare/Military	1 (1.2%)	1 (1.1%)	
Private insurance, Medicaid, and Medicare	1 (1.2%)	0 (0.0%)	
Insurance Type (version 2), *n* (%)			0.1926 ^1^
Private insurance only	40 (50.6%)	37 (40.7%)	
Medicare/Medicaid combinations	39 (49.4%)	54 (59.3%)	
Missing	2	3	
Current MyG User, *n* (%)			0.0842 ^1^
Yes	71 (87.7%)	73 (77.7%)	
No	10 (12.3%)	21 (22.3%)	
Gene, *n* (%)			0.4951 ^1^
*APOB*	27 (33.3%)	58 (61.7%)	
*LDLR*	54 (66.7%)	36 (38.3%)	

^1^ Chi-Squared *p*-value; ^2^ Wilcoxon rank sum *p*-value; ^3^ Includes Asian, Black or African American, or Prefer not to answer; ^4^ Includes Hispanic or Latino or Prefer not to answer.

**Table 2:** Proband Medical Behaviors and Outcomes.

Behavior or Outcome	Timepoint		Genetic Counseling Completed by Proband?		*p*-Value
			Yes (*n* = 80)	No (*n* = 90)	
Cardiology Appointment, *n* (%)	Pre-Disclosure (within 5 yr)		34 (42.5%)	36 (40.0%)	0.7410 ^1^
	Post- Disclosure	All Appointments	57 (71.2%)	22 (24.4%)	<0.0001 ^1^
		New Appointments	28 (35.0%)	5 (5.6%)	<0.0001 ^1^
Lipid Lowering Therapy, *n* (%)	Pre-Disclosure Prescriptions (within 1 yr)		48 (60.0%)	51 (56.7%)	0.6600 ^1^
	All Active Meds Post-Disclosure		62 (77.5%)	52 (57.8%)	0.0063 ^1^
Lipid Panel, *n* (%)	Pre-Disclosure (within 1 yr)		47 (58.8%)	52 (57.8%)	0.8979 ^1^
	Post-Disclosure		66 (82.5%)	53 (58.9%)	0.0008 ^1^
Average LDL-C Levels (mg/dL), *n* (%)	Pre-Disclosure	Median (IQR)	124.0 (99.0, 153.0)	122.0 (96.0, 152.5)	0.6913 ^2^
		Missing	4	10	
	Post- Disclosure	Median (IQR)	100.0 (64.0, 128.0)	118.0 (81.0, 150.0)	0.0473 ^2^
		Missing	14	37	
	Change in LDL-C	Median (IQR)	−13.0 (−61.0, 4.0)	−1.0 (−16.0, 17.0)	0.0054 ^2^
		Missing	17	39	
Cardiovascular Event ^3^	Pre-Disclosure		15 (18.8%)	26 (28.9%)	0.1230 ^1^
	Post- Disclosure	New Event	7 (8.8%)	3 (3.3%)	0.1341 ^1^
		Recurrent Event	13 (16.2%)	16 (17.8%)	0.7915 ^1^

^1^ Chi-Squared *p*-value; ^2^ Wilcoxon rank sum *p*-value; ^3^ Stroke, Myocardial Infarction, and/or Coronary Artery Disease.

**Table 3:** Family Communication Choice for First Degree Relatives by Proband Genetic Counseling.

First Degree Relatives: Proband Completed Genetic Counseling	Yes (*n* = 343)	No (*n* = 382)	Total (*n* = 725)	*p*-Value
Total FDRs with Strategy Selection				<0.0001 ^1^
No strategy, *n* (%)	119 (34.7%)	228 (59.7%)	347 (47.9%)	
1+ strategy, *n* (%)	224 (65.3%)	154 (40.3%)	378 (52.1%)	
FDR with 1+ Strategy, per Proband				0.0005 ^2^
Probands, *n*	64	53	117	
Mean (SD)	3.6 (1.97)	2.9 (1.44)	3.3 (1.78)	
Median (IQR)	3.0 (2.0, 4.0)	3.0 (2.0, 4.0)	3.0 (2.0, 4.0)	
Range	1.0, 9.0	1.0, 7.0	1.0, 9.0	
Strategies Selected, per FDR				0.0915 ^2^
FDR with 1+ strategy selected, *n*	224	154	378	
Mean (SD)	1.3 (0.46)	1.2 (0.41)	1.2 (0.45)	
Median (IQR)	1.0 (1.0, 2.0)	1.0 (1.0, 1.0)	1.0 (1.0, 1.0)	
Range	1.0, 3.0	1.0, 3.0	1.0, 3.0	
Type of Strategy Selections, *n*, (%)				<0.0001 ^1^
Packet Only	118 (34.4%)	115 (30.1%)	233 (32.1%)	0.2160 ^1^
Chatbot Only	38 (11.1%)	8 (2.1%)	46 (6.3%)	<0.0001 ^1^
Direct Contact Only	9 (2.6%)	2 (0.5%)	11 (1.5%)	0.0209 ^1^
Packet and Chatbot	42 (12.2%)	24 (6.3%)	66 (9.1%)	0.0053 ^1^
Packet and Direct Contact	9 (2.6%)	2 (0.5%)	11 (1.5%)	0.0209 ^1^
Chatbot and Direct Contact	6 (1.8%)	2 (0.5%)	8 (1.1%)	0.1585 ^3^
Chatbot, Direct Contact, and Packet	2 (0.6%)	1 (0.3%)	3 (0.4%)	0.6054 ^3^

^1^ Chi-Squared *p*-value; ^2^ Wilcoxon rank sum *p*-value; ^3^ Fisher’s exact *p*-value.

**Table 4:** Family Communication Choice for All Relatives by Proband Genetic Counseling.

All Relatives: Proband Completed Genetic Counseling	Yes (*n* = 1440)	No (*n* = 475)	Total (*n* = 1915)
Total Relatives with Strategy Selection			
No strategy, *n* (%)	1073 (74.5%)	301 (63.4%)	1374 (71.8%)
1+ strategy, *n* (%)	367 (25.5%)	174 (36.6%)	541 (28.2%)
Relatives with 1+ Strategy, per Proband			
Probands, *n*	65	56	121
Mean (SD)	5.9 (5.37)	3.1 (1.58)	4.6 (4.29)
Median (IQR)	5.0 (3.0, 6.0)	3.0 (2.0, 4.0)	4.0 (2.0, 5.0)
Range	1.0, 32.0	1.0, 7.0	1.0, 32.0
Strategies Selected, per Relative			
Relatives with 1+ strategy selected, *n*	367	174	541
Mean (SD)	1.2 (0.44)	1.2 (0.42)	1.2 (0.44)
Median (IQR)	1.0 (1.0, 1.0)	1.0 (1.0, 1.0)	1.0 (1.0, 1.0)
Range	1.0, 3.0	1.0, 3.0	1.0, 3.0
Type of Strategy Selections, *n*, (%)			
Packet Only	193 (13.4%)	125 (26.3%)	318 (16.6%)
Chatbot Only	73 (5.1%)	12 (2.5%)	85 (4.4%)
Direct Contact Only	18 (1.2%)	2 (0.4%)	20 (1.0%)
Packet and Chatbot	61 (4.2%)	30 (6.3%)	91 (4.8%)
Packet and Direct Contact	13 (0.9%)	2 (0.4%)	15 (0.8%)
Chatbot and Direct Contact	6 (0.4%)	2 (0.4%)	8 (0.4%)
Chatbot, Direct Contact, and Packet	3 (0.2%)	1 (0.2%)	4 (0.2%)

**Table 5:** Cascade Testing in First-Degree Relatives by Proband Genetic Counseling.

First Degree Relatives: Proband Completed Genetic Counseling	Yes (*n* = 343)	No (*n* = 382)	Total (*n* = 725)	Incidence Rate Ratio (95% CI)	*p*-Value
Cascade Testing Uptake, *n* (%)				2.25 (1.29, 3.93)	0.0043 ^1^
Cascade testing completed	93 (27.1%)	46 (12.0%)	139 (19.2%)		
Cascade testing not completed	250 (72.9%)	336 (88.0%)	586 (80.8%)		
Genetic Testing Completed Only, *n* (%)				4.45 (1.51, 13.10)	0.0066 ^1^
Yes	24 (7.0%)	6 (1.6%)	30 (4.1%)		
No	319 (93.0%)	376 (98.4%)	695 (95.9%)		
Lipid Testing Completed Only, *n* (%)				1.61 (0.82, 3.14)	0.1634 ^1^
Yes	52 (15.2%)	36 (9.4%)	88 (12.1%)		
No	291 (84.8%)	346 (90.6%)	637 (87.9%)		
Both Genetic and Lipid Testing Completed, *n* (%)				4.73 (0.88, 25.36)	0.0695 ^1^
Yes	17 (5.0%)	4 (1.0%)	21 (2.9%)		
No	326 (95.0%)	378 (99.0%)	704 (97.1%)		

^1^ Generalized Estimating Equation model for a Poisson regression.

**Table 6:** Cascade Testing in All Relatives by Proband Genetic Counseling.

All Relatives: Proband Completed Genetic Counseling	Yes (*n* = 1440)	No (*n* = 475)	Total (*n* = 1915)
Cascade Testing Uptake, *n* (%)			
Cascade testing completed	123 (8.5%)	54 (11.4%)	177 (9.2%)
Cascade testing not completed	1317 (91.5%)	421 (88.6%)	1738 (90.8%)
Genetic Testing Completed Only, *n* (%)			
Yes	39 (2.7%)	9 (1.9%)	48 (2.5%)
No	1401 (97.3%)	466 (98.1%)	1867 (97.5%)
Lipid Testing Completed Only, *n* (%)			
Yes	65 (4.5%)	40 (8.4%)	105 (5.5%)
No	1375 (95.5%)	435 (91.6%)	1810 (94.5%)
Both Genetic and Lipid Testing Completed, *n* (%)			
Yes	19 (1.3%)	5 (1.1%)	24 (1.3%)
No	1421 (98.7%)	470 (98.9%)	1891 (98.7%)

## Data Availability

The data presented in this study are available on request from the corresponding author. Data are not publicly available to protect patient privacy.
